# Effect of APOE ε4 allele on survival and fertility in an adverse environment

**DOI:** 10.1371/journal.pone.0179497

**Published:** 2017-07-06

**Authors:** Eric van Exel, Jacob J. E. Koopman, David van Bodegom, Johannes J. Meij, Peter de Knijff, Juventus B. Ziem, Caleb E. Finch, Rudi G. J. Westendorp

**Affiliations:** 1 Department of Psychiatry, VU University Medical Center/GGZinGeest, Amsterdam, the Netherlands; 2 EMGO Institute for Health and Care Research and Neuroscience Campus Amsterdam, the Netherlands; 3 Department of Gerontology and Geriatrics, Leiden University Medical Center, Leiden, the Netherlands; 4 Leyden Academy on Vitality and Ageing, Leiden, the Netherlands; 5 University of Melbourne, Melbourne Academic Center of Health, Melbourne, Australia; 6 Department of Human Genetics, Leiden University Medical Center, Leiden, the Netherlands; 7 Department of Clinical Laboratory Sciences, School of Medicine and Health Sciences, University for Development Studies, Tamale, Ghana; 8 Davis School of Gerontology and Dornsife College, Dept Biological Sciences, University of Southern California, Los Angeles, United States of America; 9 Department of Public Health, and Center for Healthy Ageing, University of Copenhagen, Copenhagen, Denmark; Duke University, UNITED STATES

## Abstract

**Background:**

The apolipoprotein-ε4 allele (*APOE-*ε4) is strongly associated with detrimental outcomes in affluent populations including atherosclerotic disease, Alzheimer’s disease, and reduced lifespan. Despite these detrimental outcomes, population frequencies of *APOE-*ε4 are high. We hypothesize that the high frequency of *APOE-*ε4 was maintained because of beneficial effects during evolution when infectious pathogens were more prevalent and a major cause of mortality. We examined a rural Ghanaian population with a high pathogen exposure for selective advantages of *APOE*-ε4, to survival and or fertility.

**Methods and findings:**

This rural Ghanaian population (n = 4311) has high levels of mortality from widespread infectious diseases which are the main cause of death. We examined whether *APOE-*ε4 was associated with survival (total follow-up time was 30,262 years) and fertility after stratifying by exposure to high or low pathogen levels. Households drawing water from open wells and rivers were classified as exposed to high pathogen levels while low pathogen exposure was classified as those drawing water from borehole wells. We found a non-significant, but positive survival benefit, i.e. the hazard ratio per *APOE-*ε4 allele was 0.80 (95% confidence interval: 0.69 to 1.05), adjusted for sex, tribe, and socioeconomic status. Among women aged 40 years and older (n = 842), *APOE*-ε4 was not associated with the lifetime number of children. However, *APOE*-ε4 was associated with higher fertility in women exposed to high pathogen levels. Compared with women not carrying an *APOE-*ε4 allele, those carrying one *APOE-*ε4 allele had on average one more child and those carrying two *APOE-*ε4 alleles had 3.5 more children (p = 0.018).

**Conclusions:**

Contrary to affluent modern-day populations, *APOE*-ε4 did not carry a survival disadvantage in this rural Ghanaian population. Moreover, *APOE*-ε4 promotes fertility in highly infectious environments. Our findings suggest that *APOE*-ε4 may be considered as evolutionarily adaptive. Its adverse associations in affluent modern populations with later onset diseases of aging further characterize *APOE*-ε4 as an example of antagonistic pleiotropy.

## Introduction

The apolipoprotein ε4 allele (*APOE* ε4 allele) it is strongly associated with detrimental outcomes to adult health including high cholesterol [1], atherosclerotic disease [2,3,4], reduced lifespan [5,6] and Alzheimer’s disease (AD) [3,7,8]. Nonetheless, *APOE* ε4 is found globally observed at population frequencies from 5 to 45%, with higher prevalence in African populations [9,10].

We and others have hypothesized that *APOE* ε4 may have beneficial or detrimental effects depending on environmental factors. Earlier in human evolution [11,12], genes such as *APOE* ε4 could have conferred a selective advantage when survival was dominated by infections, by either increasing survival, or by increasing fertility. Such adaptive value has diminished with declining infections during the past century resulting in delayed disadvantageous consequences at later ages which were on weaker selection. A prior example of beneficial or detrimental effects on survival is the sporadic Afrikaner-2 mutation in the LDL receptor gene: before 1900, the Afrikaner-2 mutation was associated with increased survival despite considerably elevated cholesterol, whereas, after 1900 as infections declined, carriers had decreased survival [13]. Thus, there can be an adaptive relationship between a genetic predisposition to elevated cholesterol levels, infectious disease, and survival. A classic example of effects of fertility dependent on environmental conditions is an isolated island opossum population which have little predation showing slower reproduction compared to a highly predated mainland population showing high reproduction [14]. Similar studies in codfish and guppies show that an environmental fluctuations causing high mortality, e.g.by overfishing [15], selects for maximization of the number of offspring [15–16].

We examined survival and fertility, and their relationship with *APOE* ε4 in a large population in rural Ghana, which is characterized by high mortality from infectious diseases [17–19].

## Materials and methods

### Ethics statement

Informed consent was obtained orally because the majority of the study participants did not read or write. A consent form was read out to the participants in their local language, explaining the purpose and procedure of the study. For the verbal autopsies, the consent form from the verbal autopsy protocol of the World Health Organization (WHO) was read out. Consent was registered in the form of a thumb print. Informed consent from children was obtained from their legal guardian, using the same procedure. The entire procedure, including the text of the consent form, was approved by the Ethical Review Committee of the Ghana Health Service, the Medical Ethical Committee of the Leiden University Medical Center in Leiden, the Netherlands, and by the local chiefs and elders in the research area.

### Study population

This study was conducted in the Garu-Tempane District in the Upper East Region of Ghana. This region is remote, rural, and among the least developed in the country. The vast majority of the inhabitants are involved in non-commercial agriculture performed by manual labor without mechanized farming or transportation. Hospital care is absent. Infectious diseases such as malaria, typhoid fever, tuberculosis, meningitis and intestinal helminths are highly endemic, whereas HIV infections range between 2 and 3% [17–24].

From 2003 through 2011, we maintained a demographic registry within a research area of 375 km^2^ comprising 32 villages. During yearly visits, we registered the name, age, sex, tribe, and location of each inhabitant. Annual migration into the research area was 2%, mostly from within the Upper East Region and surrounding areas in Togo, and 1% out of the research area. In 2007, we determined an index of socioeconomic status based on the property value of each household according to the Demographic and Health Survey method [21].

We classified inhabitants as exposed to high or low pathogen levels based on the main water source for their households. Those drawing water from open wells and rivers were classified as exposed to high pathogen levels, while those drawing water from borehole wells were classified as exposed to low pathogen levels [17]. Intestinal helminthic infections, assessed by PCR in stool samples of 611 apparently healthy individuals [20], were 1.3 times more prevalent in those drawing water from open wells and rivers (39% vs. 29%, *p* = 0.022). Mortality among the inhabitants of the research area was 17% higher (*p* = 0.003) in those drawing water from open wells and rivers vs. those drawing water from boreholes, adjusted for age, sex, tribe and socioeconomic status. Based on data from verbal autopsy [17], mortality due to infectious diseases was 20% higher in those drawing water from open wells and rivers (*p* = 0.02), while mortality due to non-infectious diseases was similar in both groups (*p* = 0.20), adjusted for age, sex, tribe and socioeconomic status.

Individuals were selected for genotyping to study longevity and fertility. Therefore, we randomly oversampled newborns, middle-aged women, and elderly from the age of 60 years onward. We preferably selected individuals included in prior phenotypic studies, which represented random selections from the population [21–25].

### Genotyping

After collection, mouth swabs were collected and stored in 2.5ml STE buffer (100mM NaCl, 10 mM Tris, 10 mM EDTA) with proteinase K (0.05 mg/ml), pronase (0.1 mg/ml) and sodium dodecylsulphate (0.5%). DNA was isolated from the buccal swabs by the commercial kit BaseClear (the Netherlands) (swabs collected 2002–2006) and KBioscience (UK) (swabs collected 2007–2010). APOE genotyping used standard TaqMan assays (Applied Biosystems, Foster City, CA, USA) and was done in the Department of Human Genetics, Leiden University Medical Center. 4975 subjects were genotyped; in 1.3% (n = 129) data on APOE genotype were incomplete. After genotyping, we rigidly excluded 535 (10.9%) individuals from the analyses. We excluded 341 individuals with a different allele at less than 10% of the loci indicated by an IBS1 (Identity by State) below 10%, which included unintentional duplicates. We excluded 199 individuals with a mismatch between the genotyped and registered sex, Five individuals were excluded because of both reasons. We have described the difficulties of field work in Ghana in a recent study [26], further explaining our exclusions.

### Lipids and inflammatory markers

Venous plasma was sampled from apparently healthy inhabitants of the research area, by random selection across 10-year age groups, except for an oversampling of women for studies on fertility [19, 24]. In these samples, levels of triglycerides and total cholesterol were measured by colorimetry (Roche Modular P800) and levels of apolipoprotein-B100 (apoB) and apolipoprotein-A1 (apoA1) by high-sensitivity immunoturbidimetry (Roche Cobas Integra). ApoA is associated with HDL cholesterol while apoB is associated with LDL cholesterol and predicts atherosclerotic disease [27]. C-reactive protein was assayed by high-sensitivity immunoturbidimetry (Roche Cobas Integra); interleukin-6 by a sandwich chemiluminescent immunoassay (Randox Evidence Investigator Biochip).

### Survival and fertility

Survival of all inhabitants was registered during the yearly follow-up from 2003 through 2011. Mortality rates were calculated as the number of deaths per 1000 person-years. Fertility was measured in two manners. Reported fertility was retrospectively determined in 2003 by interviewing women aged 40 years and older who were available and willing to participate [24,25]. Reported fertility was expressed as the total number of live births. We made significant efforts to minimize recall bias, to obtain the best estimate of the total number of offspring, by discussing the number of offspring, among all adults present in the householdincluding multiple wives. Use of contraception in this reported cohort is likely to be very low, as reported by the University of Ghana in 1988 as 0.7% for this region [28]. We, therefore, assume that when the women in this cohort were on average 23 years, in 1973, the use of contraception in this region was likely to be lower than 0.7%.

Observed fertility was registered prospectively for women of all ages during the annual follow-up from 2003 through 2011 and was expressed as the number of children that a woman had given birth to per year of follow-up [25]. It is likely that use of contraception in this cohort ranges between 15–42% [29].

### Analyses

First, the Hardy-Weinberg equilibrium of the different *APOE* alleles was tested by chi-square. Next, the number of *APOE* ε4 were used as the determinants of the levels of lipids and inflammatory markers, mortality, and fertility. Because of skewed distributions, levels of triglycerides, C-reactive protein and interleukin-6 were analyzed after logarithmic transformation and presented as geometric means. Associations of *APOE* ε4 and *APOE* ε2 with levels of lipids and inflammatory markers were assessed with an additive linear regression model, adjusted for age and socioeconomic status as continuous covariates and for sex and tribe as categorical covariates.

Mortality associations with *APOE* alleles were assessed as Kaplan-Meier survival curves. Mortality rates were assessed by Poisson regression, adjusted for socioeconomic status as a continuous covariate and sex and tribe as categorical covariates. Additionally, hazard ratios were calculated with Cox regression using the longitudinal individual survival data with age as the time variable and with left truncation to account for differences in age during follow-up.

Fertility associations of *APOE* alleles were assessed using the number of children given birth to during life and the differences in these numbers with Poisson regression, adjusted for socioeconomic status as a continuous covariate and tribe as categorical covariates. Age was included as a categorical covariate divided into 5-year age groups to account for a non-linear age pattern of fertility.

For individuals with different numbers of *APOE* ε4 or *APOE* ε2, we estimated birth rates, expressed as the number of children given birth to per year, and the differences in these rates with Poisson regression, adjusted for socioeconomic status as a continuous covariate and sex and tribe as categorical covariates. Age was again included as a categorical covariate.

All analyses were repeated after stratification of individuals exposed to high and low pathogen levels. To account for shared environmental conditions, all analyses were also clustered per household, but this did not materially change the results. The analyses used IBM SPSS version 23 and Stata/SE version 14.

### Role of the funding source

The sponsors of the study had no role in study design, data collection, primary data analysis, data interpretation, or writing of the report. The corresponding author had full access to all the data in the study and had final responsibility for the final content of the report and the decision to submit for publication.

## Results

Table 1 describes the Ghanaian study population in terms of mortality (n = 4311). Reported fertility is expressed as the number of children that women, 40 years and over had given birth to during life (n = 842). Observed fertility expressed as the number of children that a woman had given birth to per year of follow-up [17] in women aged 15 to 40 years (n = 1243) and lipids and inflammatory markers (n = 413). The frequencies of *APOE ε4 was* 14.9%. The different *APOE* alleles were not in Hardy-Weinberg equilibrium in the genotyped population (n = 4311), S1 Table.

**Table 1:** Characteristics of the Ghanaian study population.

	Lipids and inflammatory markers	Mortality	Reported fertility	Observed fertility
*n*	413	4311	842	1243
Age, years	44 (34–65)	27 (0–51)	53 (44–64)	28 (24–34)
Female sex, *n* (%)	303 (73·4)	2810 (65·2)	842 (100·0)	1243 (100·0)
Tribe, *n* (%)				
• Bimoba	312 (75·5)	2940 (68·2)	543 (64·5)	903 (72·6)
• Kusasi	93 (22·5)	1090 (25·3)	237 (28·1)	266 (21·4)
• Other	8 (1·9)	281 (6·5)	62 (7·4)	74 (6·0)
Household property, US$	1196 (688–2084)	1125 (588–1942)	1102 (515–1963)	1140 (612–2027)
Safe drinking water, *n* (%)*	308 (74·6)	3492 (81·0)	674 (80·0)	1007 (81·0)
*APOE* genotype, *n* (%)				
• ε2/ε2	12 (2·9)	111 (2·6)	20 (2·4)	35 (2·8)
• ε3/ε3	200 (48·4)	2084 (48·3)	424 (50·4)	557 (44·8)
• ε2/ε3	83 (20·1)	851 (19·7)	164 (19·5)	282 (22·7)
• ε2/ε4	26 (6·3)	214 (5·0)	43 (5·1)	63 (5·1)
• ε3/ε4	82 (19·9)	920 (21·3)	172 (20·4)	271 (21·8)
• ε4/ε4	10 (2·4)	131 (3·0)	19 (2·3)	35 (2·8)
Observed years	NA	30262	NA	10072
Deaths	NA	366	NA	NA
Number of children	NA	NA	7 (6–9)	1 (0–2)

Reported fertility was expressed as the number of children that a woman had given birth to during her life. Reported fertility was retrospectively determined in 2003 by interviewing women aged 40 years and older [19,20] Observed fertility was registered prospectively for women of all ages during the annual follow-up from 2003 through 2011 and was expressed as the number of children that a woman had given birth to per year of follow-up [12]. NA: not applicable.

* Safe drinking water, is defined as water obtained from a borehole well vs unsafe drinking water obtained from an open well, i.e. river.

**Fig 1:**
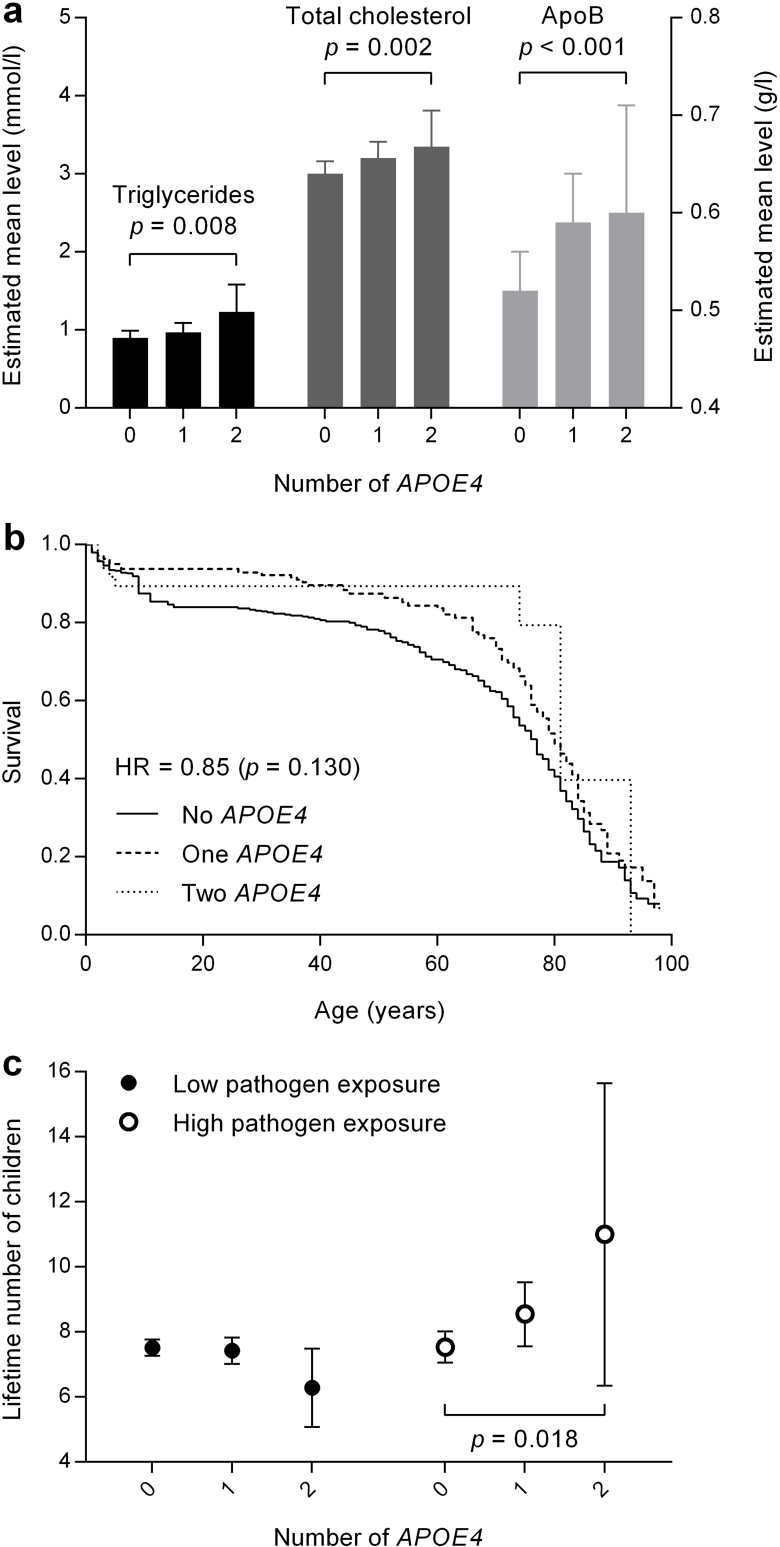
Levels of lipids, mortality and fertility per number of *APOE* ε4.

As in affluent populations, *APOE ε4* was strongly and positively associated with plasma levels of triglycerides, total cholesterol and apolipoprotein-B100 (apoB), which are closely linked to LDL cholesterol and atherosclerotic disease [27] (Table 2 and Fig 1A). Table 2 shows an allele dose for *APOE* ε4 and plasma levels of triglycerides, total cholesterol and apoB. However, *APOE ε4* (Table 2) was not associated with the inflammatory markers C-reactive protein or interleukin-6.

**Table 2:** Levels of lipids and inflammatory markers, survival, and fertility by number of *APOE* ε4 alleles.

		Number of *APOE* ε4		
	0	1	2	*p* value
**Lipids and inflammatory markers**	*n* = 295	*n* = 108	*n* = 10	
Triglycerides, mmol/l	0·90 (0·82 to 0·99)	0·97 (0·87 to 1·09)	1·23 (0·95 to 1·58)	0·008
Total cholesterol, mmol/l	3·00 (2·81 to 3·16)	3·20 (2·99 to 3·41)	3·35 (2·89 to 3·81)	0·002
Apolipoprotein-A1, g/l	1·01 (0·96 to 1·07)	1·00 (0·93 to 1·07)	0·89 (0·74 to 1·04)	0·225
Apolipoprotein-B100, g/l	0·52 (0·48 to 0·56)	0·59 (0·54 to 0·64)	0·60 (0·49 to 0·71)	< 0·001
C-reactive protein, mg/l	1·03 (0·75 to 1·42)	0·93 (0·63 to 1·36)	0·68 (0·29 to 1·59)	0·262
Interleukin-6, ng/l	1·91 (1·67 to 2·19)	1·99 (1·69 to 2·33)	2·10 (1·47 to 3·00)	0·423
**Survival**				
Mortality rate, deaths per 1000 person-years	*n* = 3046	*n* = 1134	*n* = 131	
• Overall	11·74 (10·34 to 13·15)	9·79 (7·74 to 11·84)	10·37 (3·17 to 17·56)	0·165
• Low pathogen exposure	11·23 (9·69 to 12·76)	9·67 (7·45 to 11·90)	10·17 (2·62 to 17·71)	0·311
• High pathogen exposure	13·96 (10·51 to 17·40)	10·15 (5·01 to 15·29)	10·97 (0·00 to 32·58)	0·285
**Reported fertility**				
Number of children, *n*	*n* = 608	*n* = 215	*n* = 19	
• Overall	7·52 (7·30 to 7·74)	7·60 (7·23 to 7·97)	6·85 (5·66 to 8·04)	0·819
• Low pathogen exposure	7·52 (7·27 to 7·76)	7·42 (7·02 to 7·82)	6·28 (5·07 to 7·48)	0·202
• High pathogen exposure	7·53 (7·05 to 8·01)	8·55 (7·56 to 9·53)	11·00 (6·34 to 15·65)	0·018
**Observed fertility**				
Birth rate, children per person-year	*n* = 874	*n* = 334	*n* = 35	
• Overall	0·96 (0·90 to 1·03)	0·92 (0·82 to 1·02)	0·99 (0·65 to 1·33)	0·641
• Low pathogen exposure	0·97 (0·90 to 1·05)	0·89 (0·78 to 1·00)	0·99 (0·61 to 1·38)	0·371
• High pathogen exposure	0·92 (0·77 to 1·06)	1·04 (0·78 to 1·30)	0·98 (0·25 to 1·72)	0·460

Lipids and inflammatory markers are given as means or geometric means with 95% confidence intervals. Apolipoprotein-A1 is associated with HDL cholesterol, while apolipoprotein-B100 is associated with LDL cholesterol. Low pathogen exposure is defined as water from borehole wells, high pathogen exposure is water from relatively unsafe sources with high levels of pathogens, such as rivers and open wells. Measures of survival and fertility are given with 95% confidence intervals. Lipids, inflammatory markers and mortality measures were adjusted for age, sex, tribe and socioeconomic status. Fertility measures were adjusted for age, tribe and socioeconomic status.

Contrary to affluent populations, *APOE ε4* was not associated with a survival disadvantage.

The total follow-up time in 4311 genotyped subjects was 30.262 years with a mean follow-up of 7 years and observed a non-significant but positive survival trend in those who were carrying one or two APOE ε4 alleles (Table 2, p = 0.165). There was no dose-response association between increasing number of *APOE* ε4 and survival (Fig 1B). The hazard ratio per *APOE* ε4 allele was 0.80 (95% confidence interval: 0.69 to 1.05), p = 0.130, adjusted for sex, tribe, and socioeconomic status. The *APOE* ε2 allele was not associated with survival (S2 Table).

Some Ghanaian villagers used water drawn from relatively unsafe sources with high levels of pathogens, such as rivers and open wells, while others drew water from relatively cleaner borehole wells with low levels of pathogens. These water sources have been located in the area independent of the household characteristics [17], i.e. social economical status or tribe. We repeated our analyses after stratification for this difference in pathogen exposure. There was still no association between *APOE* ε4 and survival after stratification by water source as a proxy for pathogen exposure (Table 2).

Next, we analyzed the association of *APOE ε4* with fertility as reported by women aged 40 years and older. Among all women, *APOE ε4* was not associated with the lifetime number of offspring (Table 2). However, when stratified by high and low pathogen exposure, fertility was higher in association with *APOE* ε4 allele dose. Moreover, the number of offspring increased with an increasing number of *APOE* ε4 in women exposed to high pathogen levels, in adjusted analysis for maternal age, tribe and socioeconomic status (Table 2 and Fig 1C) and in unadjusted analysis (Panel C in S1 Fig).

Relative to the non-carriers, those carrying one APOE ε4 had on average one more child and those carrying two APOE ε4 had 3.5 more children (Table 2, Fig 1C). In contrast, with low pathogen exposure, APOE ε4 was not associated with the number of offspring (Table 2, Fig 1C). We analyzed the distribution of the number of APOE- ε4 alleles in relation to the pathogen exposure, to determine whether our finding on fertility was possibly due to a skewed distribution of APOE ε4, However, the APOE- ε4 allele frequency is similarly distributed in both groups. The APOE- ε4 allele frequency is 12.5% in the high pathogen exposure group vs. 15.7% in the lower exposure group (S3 Table). The *APOE* ε2 allele did not show an association with reported fertility (S2 Table).

We examined the association of *APOE* with fertility as observed during follow-up in women aged 15 through 40 years. Neither the *APOE* ε4 allele (Table 2) nor the *APOE* ε2 allele (S2 Table) were associated with birth rates.

Finally, we explored the association of the genotypes of APOE ε3/ε4 (frequency in the studied population 21.3%), ε2/ε4 (frequency of 5.0%),ε2/ε3 (19.7%) and those without an *APOE-ε4* allele to those carrying an *APOE*-ε4 allele, i.e. *APOE*-ε4, heterozygotes and homozygotes combined with either survival (S4 Table and S5 Table) or fertility (S6 Table). The findings agree with our other findings that showed the *APOE*-ε4 allele did not confer a survival disadvantage in this rural Ghanaian population. Moreover, all analyses revealed a trend with improved survival in *APOE*-ε3/ε4, ε2/ε4 and those without an *APOE*-ε4 allele compared to those carrying an *APOE* -ε4 allele, i.e. *APOE* -ε4, heterozygotes and homozygotes combined carriers. Our additional analysis also confirmed our finding on fertility in those exposed to high pathogen levels, as shown in Table 2 that those without an *APOE* -ε4 allele compared to those carrying an *APOE* -ε4 allele, i.e. one or two APOE ε4, compared with those not carrying *APOE-* ε4, had on average one more child, p = 0.033 (S7 Table). A similar trend was revealed with improved reported fertility in *APOE* -ε3/ε4 and ε2/ε4 carriers using unsafe drinking water sources.

Moreover, we studied the number of offspring in *APOE-*ε4 homozygotes compared to APOE ε4 heterozygotes, in environments with high pathogen levels (S8 Table), suggesting that APOE ε4 homozygotes compared to *APOE-*ε4 heterozygotes, had more offspring.

## Discussion

The effect of the *APOE* genotype on survival and fertility was studied in a large population in rural Ghana in which mortality across the lifespan is dominated by infectious diseases. We report three novel observations: first, *APOE ε4* in populations living in an adverse environment was positively associated with plasma levels of cholesterol and triglycerides, as in affluent populations; second, *APOE ε4* did not act a survival disadvantage, in contrast to affluent populations with low pathogen exposure; and third, in women exposed to higher pathogen levels *APOE ε4* was associated with greater fertility and survival of offspring.

### APOE ε4, lipids, and survival

The observed *APOE* ε4, associations with plasma cholesterol and triglycerides in this Ghanaian study population, is of interest in two regards. First, the association of *APOE ε4* with elevated blood lipids has been documented for affluent populations with low levels of infections only. The association of *APOE ε4* with elevated cholesterol, triglycerides and apoB holds, despite the remarkable low levels of plasma cholesterol, triglycerides and apoB found in the Ghanaian population, where infections are highly prevalent. A similarly low cholesterol level was seen in the Tsimane, indigenous farmer-foragers of Bolivia, who also suffer high mortality from a high pathogen load [30]. Second, while *APOE ε4* was associated with higher levels of triglycerides and cholesterol, in contrast to affluent populations with a low pathogen exposure, *APOE ε4* did not have a detrimental and possibly a beneficial effect on survival in this Ghanaian population with its high pathogen exposure. This is consistent with our earlier findings on the Afrikaner-2 mutation, which elevated cholesterol, but also conferred a survival benefit in Europe before 1900 in an endemically infected population [13], whereas, after 1900 as infections declined, this mutation decreased survival. We conclude that contemporary observations in affluent societies showing the late life detrimental effects of *APOE* ε4 are best considered as an example of specific gene-environmental interaction [1–8].

### APOE ε4 and fertility

In addition to its relative survival benefit, *APOE* ε4 was associated with higher fertility. In women exposed to high pathogen levels, *APOE* ε4 was associated with an increased number of reported offspring. These findings show the advantage of determining fertility in a population with a low level use of contraception, i.e. women from our reported/retrospective cohort on fertility. Among these women who largely did not use contraception, fertility was associated with *APOE* ε4, in those exposed to high pathogen levels. These differences in fertility in relation to *APOE* ε4 however, are not apparent in a population were contraception use is more widespread, i.e. our prospective cohort. The effects of *APOE* ε4 on fecundity seem harder to detect where contraception influences fertility.

Our observations on *APOE* ε4 and fertility extends findings from rural Ecuador, where *APOE ε4* carrying women had more offspring [31]· Moreover, young regularly menstruating women with *APOE ε4* in rural and urban Poland have higher levels of progesterone in the luteal phase, suggesting a higher fecundability [32]. However, the findings from these two studies are based on relatively small samples.

Finally, the data on fertility could be hampered. One could argue that the data for *APOE* ε4 homozygotes is influenced due to the low sample size, n = 19. We therefore did two additional anaysis, first we showed that the *APOE* ε4 allele frequency was similar in those exposed to the high pathogen levels vs those exposed to low pathogen levels (S3 Table). Second we showed that our results remained similar when we compared those without *APOE* ε4 to those with one or two *APOE* ε4. In subjects in environments with high pathogen exposure, those with one or two APOE ε4 had more offspring compared to those without an APOE ε4, These additional analysis underscore the idea that at least presence of one *APOE* ε4, contributes to increased fertility in environments with high pathogen exposure.

Together, our empirical findings support the hypothesis that *APOE ε4* was maintained by Darwinian adaptive value in environments with a high pathogen exposure, where reproduction has been favoured and where limited survival to later ages would have delayed detrimental effects of *APOE ε4* that include atherosclerotic disease [1–4]^,^ Alzheimer’s disease [1,3,5,6], and accelerated cognitive decline. Moreover, the detrimental effects of *APOE ε4* may be less apparent in environments with a high pathogen exposure such as rural Ghana, wherein, due to limited access to nutrition and daily demanding physical activity, ischemic atherosclerotic disease is relatively rare [19]. We and others have hypothesized that the *APOE* ε4 allele may have beneficial or detrimental effects depending on environmental factors. According to the antagonistic pleiotropy hypothesis [11,12], earlier in human evolution, the *APOE* ε4 allele conferred a selective advantage by increasing pathogen resistance when mortality was dominated by infections, with delayed adverse consequences at later ages which were on weaker selection. Such adaptive value has diminished with declining infections during the past century.

Lastly, we consider the *APOE* ε2 allele, which was 1% more common than expected in the Ghanaian study population, consistent with its relatively high prevalence in African populations [9,10]. In contrast to the *APOE* ε4 allele, the *APOE* ε2 allele did not confer benefits to either survival or fertility. We suggest that the potential effects of the *APOE* ε2 allele are smaller than those of the *APOE* ε4 allele, which may require a larger population to be detected or could be a result of genetic drift rather than natural selection. Alternatively, that the potential effects of the *APOE* ε2 allele may be dependent on environmental conditions other than pathogen exposure.

## Conclusions

We show novel findings that *APOE ε4* did not negatively affect survival in a contemporary environment dominated by infectious diseases. Additionally, *APOE ε4* was associated with a higher number of offspring in women exposed to high pathogen levels. The latter findings suggest the Darwinian selective advantage of *APOE ε4* by enhancing fertility under adverse environmental conditions of a high pathogen exposure. Such prior associations with pathogen resistance may explain its persistence in affluent populations with low pathogen exposure, despite its late life deleterious effects.

## Supporting information

S1 Fig Levels of lipids, mortality and fertility per number of *APOE* ε4, unadjusted analysis. (TIFF)

S1 Table Frequencies of APOE genotypes in the Ghanaian study population. (DOCX)

S2 Table Frequency of APOE genotypes among individuals exposed to high or low pathogen levels, i.e those having access to unsafe or safe water. (DOCX)

S3 Table Levels of lipids and inflammatory markers, survival, and fertility by number of APOE ε2. (DOCX)

S4 Table Association of the genotypes of APOE ε3/ε4,ε2/ε4 and ε2/ε3 with survival. (DOCX)

S5 Table Association of the genotypes of APOE ε3/ε4,ε2/ε4 and ε2/ε3 with survival. (DOCX)

S6 Table Association of the genotypes of APOE ε3/ε4,ε2/ε4 and ε2/ε3 fertility. (DOCX)

S7 Table Reported and observed fertility in individuals carrying one or two APOE ε4 compared with those not carrying APOE ε4. (DOCX)

S8 Table Comparison of reported and observed fertility in individuals carrying one or two APOE ε4. (DOCX)
