# Supplementary figures and images for: Effect of APOE ε4 allele on survival and fertility in an adverse environment
Source: PLoS One. 2017 Jul 6;12(7):e0179497. doi: 10.1371/journal.pone.0179497 (PMC5500260; doi:10.1371/journal.pone.0179497)

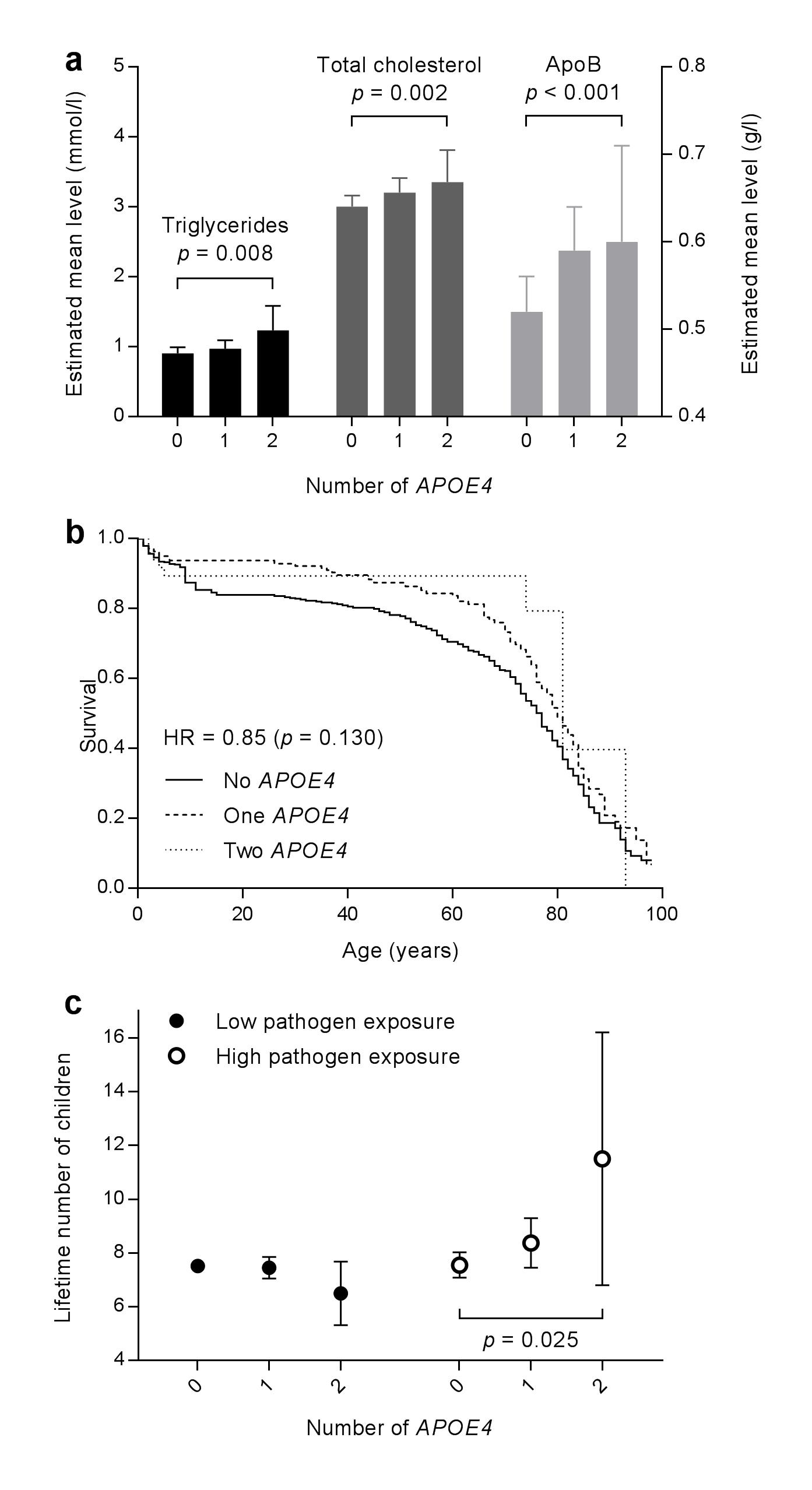

Supplement: S1 Fig — (TIFF) [file pone.0179497.s001.tiff]
