# Supplementary material for: Effect of APOE ε4 allele on survival and fertility in an adverse environment
Source: PLoS One. 2017 Jul 6;12(7):e0179497. doi: 10.1371/journal.pone.0179497 (PMC5500260; doi:10.1371/journal.pone.0179497)
Supplement: S1 Table — (DOCX) [file pone.0179497.s002.docx]

**Supplemental table 1. Frequencies of *APOE* genotypes in the Ghanaian study population**

|  | **Observed** | |  | **Expected** | |
| --- | --- | --- | --- | --- | --- |
|  | ***n*** | **%** |  | ***n*** | **%** |
| ε2/ε2 | 111 | 2·6 |  | 96 | 2·2 |
| ε3/ε3 | 2084 | 48·3 |  | 2045 | 47·5 |
| ε2/ε3 | 851 | 19·7 |  | 887 | 20·6 |
| ε2/ε4 | 214 | 5·0 |  | 208 | 4·8 |
| ε3/ε4 | 920 | 21·3 |  | 962 | 22·3 |
| ε4/ε4 | 131 | 3·0 |  | 113 | 2·6 |
| All | 4311 | 100·0 |  | 4311 | 100·0 |

Chi-square test for Hardy-Weinberg equilibrium: *p*=0·0257.
