# Supplementary material for: Effect of APOE ε4 allele on survival and fertility in an adverse environment
Source: PLoS One. 2017 Jul 6;12(7):e0179497. doi: 10.1371/journal.pone.0179497 (PMC5500260; doi:10.1371/journal.pone.0179497)
Supplement: S2 Table — (DOCX) [file pone.0179497.s003.docx]

**Supplementary Table 2. Levels of lipids and inflammatory markers, survival, and fertility by number of APOE ε2**

|  | **Number of *APOE* ε2** | | |  |
| --- | --- | --- | --- | --- |
|  | **0** | **1** | **2** | ***p* value** |
| **Lipids and inflammatory markers** | *n* = 292 | *n* = 109 | *n* = 12 |  |
| Triglycerides, mmol/l | 0.94 (0.85 to 1.03) | 0.87 (0.77 to 0.98) | 1.05 (0.83 to 1.34) | 0.451 |
| Total cholesterol, mmol/l | 3.12 (2.95 to 3.30) | 2.79 (2.58 to 3.00) | 2.53 (2.11 to 2.95) | < 0.001 |
| Apolipoprotein-A1, g/l | 0.99 (0.94 to 1.05) | 1.05 (0.98 to 1.12) | 1.16 (1.01 to 1.30) | 0.002 |
| Apolipoprotein-B100, g/l | 0.57 (0.53 to 0.61) | 0.45 (0.41 to 0.50) | 0.24 (0.14 to 0.35) | < 0.001 |
| C-reactive protein, mg/l | 1.03 (0.75 to 1.42) | 0.91 (0.62 to 1.33) | 0.85 (0.39 to 1.87) | 0.333 |
| Interleukin-6, ng/l | 1.92 (1.68 to 2.20) | 1.91 (1.63 to 2.25) | 2.32 (1.67 to 3.23) | 0.552 |
| **Survival** |  |  |  |  |
| Mortality rate, deaths per 1000 person-years | *n* = 3135 | *n* = 1065 | *n* = 111 |  |
| Overall | 11.36 (10.01 to 12.71) | 10.86 (8.54 to 13.17) | 8.63 (2.23 to 15.02) | 0.493 |
| Low pathogen exposure | 10.88 (9.42 to 12.34) | 10.61 (8.02 to 13.19) | 8.59 (1.71 to 15.47) | 0.644 |
| High pathogen exposure | 13.56 (10.07 to 17.04) | 11.93 (6.64 to 17.22) | 7.69 (0.00 to 22.80) | 0.490 |
| **Reported fertility** |  |  |  |  |
| Lifetime number of children, *n* | *n* = 615 | *n* = 207 | *n* = 20 |  |
| Overall | 7.54 (7.32 to 7.76) | 7.47 (7.10 to 7.85) | 7.68 (6.47 to 8.89) | 0.898 |
| Low pathogen exposure | 7.48 (7.23 to 7.72) | 7.42 (7.01 to 7.83) | 7.41 (5.99 to 8.82) | 0.810 |
| High pathogen exposure | 7.81 (7.32 to 8.30) | 7.69 (6.72 to 8.66) | 8.08 (5.81 to 10.35) | 0.994 |
| **Observed fertility** |  |  |  |  |
| Birth rate, children per person-year | *n* = 874 | *n* = 334 | *n* = 35 |  |
| Overall | 0.95 (0.89 to 1.02) | 0.95 (0.84 to 1.05) | 0.95 (0.65 to 1.25) | 0.908 |
| Low pathogen exposure | 0.96 (0.89 to 1.03) | 0.93 (0.82 to 1.05) | 0.93 (0.60 to 1.25) | 0.696 |
| High pathogen exposure | 0.92 (0.77 to 1.07) | 1.04 (0.79 to 1.28) | 0.94 (0.26 to 1.63) | 0.498 |

Lipids and inflammatory markers are given as means or geometric means with 95% confidence intervals. Apolipoprotein-A1 is associated with HDL cholesterol, while apolipoprotein-B100 is associated with LDL cholesterol. Low pathogen exposure is defined as water from borehole wells, high pathogen exposure is water from relatively unsafe sources with high levels of pathogens, such as rivers and open wells. Measures of survival and fertility are given with 95% confidence intervals. Differences in lipids and inflammatory markers and reported fertility were tested with linear regression. Differences in survival and observed fertility were tested with Poisson regression. Lipids, inflammatory markers and mortality measures were adjusted for age, sex, tribe and socioeconomic status. Fertility measures were adjusted for age, tribe and socioeconomic status.
