# Supplementary material for: Effect of APOE ε4 allele on survival and fertility in an adverse environment
Source: PLoS One. 2017 Jul 6;12(7):e0179497. doi: 10.1371/journal.pone.0179497 (PMC5500260; doi:10.1371/journal.pone.0179497)
Supplement: S3 Table — (DOCX) [file pone.0179497.s004.docx]

**Supplemental table 3.** Frequency of APOE genotypes among individuals exposed to high or low pathogen levels, i.e those having access to unsafe or safe water.

|  | Level of pathogen exposure | |
| --- | --- | --- |
| APOE- ε genotype | High  (n=168) | Low  (n=674) |
| ε2/ε2 (%) | 6 (3.6) | 14 (2.1) |
| ε2/ε3 (%) | 25 (14.9) | 139 (20.6) |
| ε2/ε4 (%) | 7 (4.2) | 36 (5.3) |
| ε3/ε3 (%) | 97 (57.7) | 327 (48.5) |
| ε3/ε4 (%) | 31 (18.5) | 141 (20.9) |
| ε4/ε4 (%) | 2 (1.2) | 17 (2.5) |
