# Supplementary material for: Effect of APOE ε4 allele on survival and fertility in an adverse environment
Source: PLoS One. 2017 Jul 6;12(7):e0179497. doi: 10.1371/journal.pone.0179497 (PMC5500260; doi:10.1371/journal.pone.0179497)
Supplement: S4 Table — (DOCX) [file pone.0179497.s005.docx]

**Supplemental Table 4a** Mortality rate in APOE ε3/ε4 compared to all other APOE genotypes

|  | APOE ε3/ε4 | Other APOE genotypes | P value |
| --- | --- | --- | --- |
| Overall Mortality rate* | 10.23 | 11.45 | 0.387 |
| Mortality rate and pathogen exposure levels |  |  |  |
| High | 11.05 | 13.41 | 0.541 |
| Low | 10.07 | 10.97 | 0.551 |

* Mortality rate, deaths per 1000 person years. Differences in mortality were tested with Poisson regression and adjusted for age, sex, tribe and socioeconomic status.

**Supplemental Table 4b** Mortality rate in APOE ε2/ε4 compared to all other APOE genotypes

|  | APOE ε2/ε4 | Other APOE genotypes | P value |
| --- | --- | --- | --- |
| Overall Mortality rate | 7.83 | 11.35 | 0.189 |
| Mortality rate and pathogen exposure levels |  |  |  |
| High | 7.47 | 13.37 | 0.329 |
| Low | 7.74 | 10.91 | 0.286 |

* Mortality rate, deaths per 1000 person years. Differences in mortality were tested with Poisson regression and adjusted for age, sex, tribe and socioeconomic status.

**Supplemental Table 4c** Mortality rate in APOE ε2/ε3 compared to all other APOE genotypes

|  | APOE ε2/ε3 | Other APOE genotypes | P value |
| --- | --- | --- | --- |
| Overall Mortality rate | 11.67 | 11.06 | 0.682 |
| Mortality rate and pathogen exposure levels |  |  |  |
| High | 13.29 | 12.91 | 0.915 |
| Low | 11.38 | 10.62 | 0.647 |

* Mortality rate, deaths per 1000 person years. Differences in mortality were tested with Poisson regression and adjusted for age, sex, tribe and socioeconomic status.

**Supplemental Table 4d** Mortality rate in APOE ε3/ε4 compared to APOE ε3/ε3 carriers

|  | APOE ε3/ε4 | APOE ε3/ε3 | P value |
| --- | --- | --- | --- |
| Overall Mortality rate | 10.42 | 12.14 | 0.266 |
| Mortality rate and pathogen exposure levels |  |  |  |
| High | 11.06 | 14.64 | 0.392 |
| Low | 10.27 | 11.55 | 0.439 |

* Mortality rate, deaths per 1000 person years. Differences in mortality were tested with Poisson regression and adjusted for age, sex, tribe and socioeconomic status.

**Supplemental Table 4e** Mortality rate in APOE ε2/ε4 compared to APOE ε3/ε3 carriers

|  | APOE ε2/ε4 | APOE ε3/ε3 | P value |
| --- | --- | --- | --- |
| Overall Mortality rate | 7.90 | 12.02 | 0.143 |
| Mortality rate and pathogen exposure levels |  |  |  |
| High | 7.61 | 15.43 | 0.247 |
| Low | 7.70 | 11.35 | 0.235 |

* Mortality rate, deaths per 1000 person years. Differences in mortality were tested with Poisson regression and adjusted for age, sex, tribe and socioeconomic status.

**Supplemental Table 4f** Mortality rate in APOE ε2/ε3 compared APOE ε3/ε3 carriers

|  | APOE ε2/ε3 | APOE ε3/ε3 | P value |
| --- | --- | --- | --- |
| Overall Mortality rate | 11.72 | 11.97 | 0.879 |
| Mortality rate and pathogen exposure levels |  |  |  |
| High | 13.93 | 15.00 | 0.797 |
| Low | 11.32 | 11.21 | 0.955 |

* Mortality rate, deaths per 1000 person years. Differences in mortality were tested with Poisson regression and adjusted for age, sex, tribe and socioeconomic status.

**Supplemental Table 4g** Mortality rate in individuals carrying one or two APOE ε4 compared with those not carrying APOE ε4

|  | No APOE ε4 | One or two APOE ε4 | P value |
| --- | --- | --- | --- |
| Overall Mortality rate | 11.74 | 9.84 | 0.136 |
| Mortality rate and pathogen exposure levels |  |  |  |
| High | 13.96 | 10.20 | 0.263 |
| Low | 11.23 | 9.71 | 0.273 |

* Mortality rate, deaths per 1000 person years. Differences in mortality were tested with Poisson regression and adjusted for age, sex, tribe and socioeconomic status.
