# Supplementary material for: Effect of APOE ε4 allele on survival and fertility in an adverse environment
Source: PLoS One. 2017 Jul 6;12(7):e0179497. doi: 10.1371/journal.pone.0179497 (PMC5500260; doi:10.1371/journal.pone.0179497)
Supplement: S5 Table — (DOCX) [file pone.0179497.s006.docx]

**Supplemental Table 5** Survival, determined with Hazard ratio’s

|  | Overall  Hazard ratio | Hazard Ratio  Pathogen exposure levels  High Low | |  |
| --- | --- | --- | --- | --- |
| APOE ε3/ε4 vs other genotypes | 0.9 (p value=0.4) | 0.94 (p value=0.7) | 0.83 (p value =0.6) | |
| APOE ε2/ε4 vs other genotypes | 0.56 (p value=0.2) | 0.69 (p value=0.3) | 0.56 (p value=0.3) | |
| APOE ε2/ε3 vs other genotypes | 1.1 (p value=0.4) | 1.12 (p value=0.4) | 1.03 (p value=0.9) | |
| APOE ε3/ε4 vs APOE ε3/ε3 | 0.90 (p value=0.4) | 0.94 (p value=0.7) | 0.83 (p value=0.6) | |
| APOE ε2/ε4 vs APOE ε3/ε3 | 0.65 (p value=0.1) | 0.66 (p value=0.2) | 0.46 (p value=0.2) | |
| APOE ε2/ε3 vs APOE ε3/ε3 | 1.02 (p value=0.9) | 1.07 (p value=0.7) | 0.98 (p value=0.9) | |
| Carrier of one APOE e4 vs other genotypes | 0.84 (p=0.2) | 0.74 (p=0.3) | 0.87 (p=0.3) | |
| Carrier of one APOE e4 vs APOE e3/e3 | 0.83 (p=0.1) | 0.70 (p=0.2) | 0.87 (p=0.3) | |
| Carrier of one or two APOE e4 vs other genotypes | 0.83 (p=0.1) | 0.73 (p=0.3) | 0.87 (p=0.3) | |
| Carrier of one or two APOE e4 vs APOE e3/e3 | 0.82 (p=0.1) | 0.70 (p=0.2) | 0.86 (p=0.3) | |

Hazard ratios were calculated with Cox regression, adjustments were made for age, sex, tribe and socioeconomic status.
