# Supplementary material for: Effect of APOE ε4 allele on survival and fertility in an adverse environment
Source: PLoS One. 2017 Jul 6;12(7):e0179497. doi: 10.1371/journal.pone.0179497 (PMC5500260; doi:10.1371/journal.pone.0179497)
Supplement: S6 Table — (DOCX) [file pone.0179497.s007.docx]

**Supplemental Table 6a** Reported and observed fertility in APOE ε3/ε4 compared to all other APOE genotypes

|  | APOE ε3/ε4 | Other APOE genotypes | P value |
| --- | --- | --- | --- |
| Overall Reported fertility | 7.53 | 7.53 | 0.997 |
| Reported fertility and pathogen exposure levels |  |  |  |
| High | 8.56 | 7.63 | 0.119 |
| Low | 7.33 | 7.50 | 0.511 |
|  |  |  |  |
| Overall Observed fertility | 0.93 | 0.96 | 0.747 |
| Observed fertility and pathogen exposure levels |  |  |  |
| High | 1.05 | 0.93 | 0.445 |
| Low | 0.91 | 0.96 | 0.481 |

Differences in fertility were tested with Poisson regression and adjusted for age, tribe and socioeconomic status.

**Supplemental Table 6b** Reported and observed fertility in APOE ε2/ε4 compared to all other APOE genotypes

|  | APOE ε2/ε4 | Other APOE genotypes | P value |
| --- | --- | --- | --- |
| Overall Reported fertility | 7.90 | 7.51 | 0.363 |
| Reported fertility and pathogen exposure levels |  |  |  |
| High | 8.42 | 7.77 | 0.556 |
| Low | 7.79 | 7.44 | 0.458 |
|  |  |  |  |
| Overall Observed fertility | 0.86 | 0.96 | 0.411 |
| Observed fertility and pathogen exposure levels |  |  |  |
| High | 1.01 | 0.95 | 0.813 |
| Low | 0.81 | 0.96 | 0.286 |

Differences in fertility were tested with Poisson regression and adjusted for age, tribe and socioeconomic status.

**Supplemental Table 6c** Reported and observed fertility in APOE ε2/ε3 compared to all other APOE genotypes

|  | APOE ε2/ε3 | Other APOE genotypes | P value |
| --- | --- | --- | --- |
| Overall Reported fertility | 7.36 | 7.57 | 0.385 |
| Reported fertility and pathogen exposure levels |  |  |  |
| High | 7.32 | 7.50 | 0.512 |
| Low | 7.49 | 7.85 | 0.554 |
|  |  |  |  |
| Overall Observed fertility | 0.97 | 0.95 | 0.756 |
| Observed fertility and pathogen exposure levels |  |  |  |
| High | 1.05 | 0.93 | 0.438 |
| Low | 0.96 | 0.95 | 0.826 |

Differences in fertility were tested with Poisson regression and adjusted for age, tribe and socioeconomic status.

**Supplemental Table 6d** Reported and observed fertility in APOE ε3/ε4 compared to APOE ε3/ε3 carriers

|  | APOE ε3/ε4 | APOE ε3/ε3 | P value |
| --- | --- | --- | --- |
| Overall Reported fertility | 7.53 | 7.58 | 0.814 |
| Reported fertility and pathogen exposure levels |  |  |  |
| High | 8.53 | 7.45 | 0.082 |
| Low | 7.33 | 7.62 | 0.302 |
|  |  |  |  |
| Overall Observed fertility | 0.92 | 0.94 | 0.756 |
| Observed fertility and pathogen exposure levels |  |  |  |
| High | 1.05 | 0.84 | 0.219 |
| Low | 0.90 | 0.97 | 0.407 |

Differences in fertility were tested with Poisson regression and adjusted for age, tribe and socioeconomic status.

**Supplemental Table 6e** Reported and observed fertility in APOE ε2/ε4 compared to APOE ε3/ε3 carriers

|  | APOE ε2/ε4 | APOE ε3/ε3 | P value |
| --- | --- | --- | --- |
| Overall Reported fertility | 7.89 | 7.58 | 0.485 |
| Reported fertility and pathogen exposure levels |  |  |  |
| High | 8.87 | 7.47 | 0.226 |
| Low | 7.76 | 7.60 | 0.752 |
|  |  |  |  |
| Overall Observed fertility | 0.84 | 0.95 | 0.401 |
| Observed fertility and pathogen exposure levels |  |  |  |
| High | 1.04 | 0.86 | 0.522 |
| Low | 0.79 | 0.97 | 0.225 |

Differences in fertility were tested with Poisson regression and adjusted for age, tribe and socioeconomic status.

**Supplemental Table 6f** Reported and observed fertility in APOE ε2/ε3 compared to APOE ε3/ε3 carriers

|  | APOE ε2/ε3 | APOE ε3/ε3 | P value |
| --- | --- | --- | --- |
| Overall Reported fertility | 7.36 | 7.57 | 0.411 |
| Reported fertility and  pathogen exposure levels |  |  |  |
| High | 7.43 | 7.55 | 0.846 |
| Low | 7.33 | 7.59 | 0.349 |
| Overall Observed fertility | 0.96 | 0.95 |  |
| Observed fertility and  pathogen exposure levels |  |  | 0.936 |
| High | 1.09 | 0.84 | 0.152 |
| Low | 0.95 | 0.97 | 0.783 |

Differences in fertility were tested with Poisson regression and adjusted for age, tribe and socioeconomic status.
