# Supplementary material for: Effect of APOE ε4 allele on survival and fertility in an adverse environment
Source: PLoS One. 2017 Jul 6;12(7):e0179497. doi: 10.1371/journal.pone.0179497 (PMC5500260; doi:10.1371/journal.pone.0179497)
Supplement: S7 Table — (DOCX) [file pone.0179497.s008.docx]

**Supplemental Table 6g** Reported and observed fertility in individuals carrying one or two APOE ε4 compared with those not carrying APOE ε4

|  | No APOE ε4 | One or two APOE ε4 | P value |
| --- | --- | --- | --- |
| Overall reported fertility | 7.52 | 7.54 | 0.923 |
| Reported fertility and pathogen exposure levels |  |  |  |
| High | 7.53 | 8.68 | 0.033 |
| Low | 7.52 | 7.32 | 0.402 |
|  |  |  |  |
| Overall observed fertility | 0.96 | 0.93 | 0.540 |
| Observed fertility and pathogen exposure levels |  |  |  |
| High | 0.92 | 1.04 | 0.408 |
| Low | 0.97 | 0.90 | 0.273 |

Differences in fertility were tested with Poisson regression and adjusted for age, tribe and socioeconomic status.
