# Supplementary material for: Effect of APOE ε4 allele on survival and fertility in an adverse environment
Source: PLoS One. 2017 Jul 6;12(7):e0179497. doi: 10.1371/journal.pone.0179497 (PMC5500260; doi:10.1371/journal.pone.0179497)
Supplement: S8 Table — (DOCX) [file pone.0179497.s009.docx]

**Supplemental Table** Comparison of **r**eported and observed fertility in individuals carrying one or two APOE ε4

|  | one APOE ε4 | two APOE ε4 | P value |
| --- | --- | --- | --- |
| Overall reported fertility | 7.59 (n=217) | 6.83 (n=20) | 0.258 |
| Reported fertility and pathogen exposure levels |  |  |  |
| High | 8.35 (n=38) | 11.95 (n=2) | 0.169 |
| Low | 7.42 (n=179) | 6.39 (n=18) | 0.109 |
|  |  |  |  |
| Overall observed fertility | 0.92 (n=341) | 1.02 (n=35) | 0.561 |
| Observed fertility and pathogen exposure levels |  |  |  |
| High | 0.98 (n=64) | 1.01 (n=7) | 0.939 |
| Low | 0.90 (n=274) | 1.04 (n=28) | 0.499 |

Differences in fertility were tested with Poisson regression and adjusted for age, tribe and socioeconomic status.
